# Teleworking, Parenting Stress, and the Health of Mothers and Fathers

**DOI:** 10.1001/jamanetworkopen.2023.41844

**Published:** 2023-11-03

**Authors:** John James Parker, Craig F. Garfield, Clarissa D. Simon, Anne Bendelow, Marie E. Heffernan, Matthew M. Davis, Kristin Kan

**Affiliations:** 1 Division of Advanced General Pediatrics and Primary Care, Department of Pediatrics, Ann & Robert H. Lurie Children’s Hospital of Chicago, Northwestern University, Chicago, Illinois; 2 Division of General Internal Medicine and Geriatrics, Northwestern University Feinberg School of Medicine, Chicago, Illinois; 3 Department of Pediatrics, Ann & Robert H. Lurie Children’s Hospital of Chicago, Northwestern University Feinberg School of Medicine, Chicago, Illinois; 4 Family and Child Health Innovations Program, Smith Child Health Outcomes, Research and Evaluation Center, Ann & Robert H. Lurie Children’s Hospital of Chicago, Chicago, Illinois; 5 Smith Child Health Outcomes, Research, and Evaluation Center, Stanley Manne Children’s Research Institute, Ann & Robert H. Lurie Children’s Hospital of Chicago, Chicago, Illinois; 6 Data Analytics and Reporting, Ann & Robert H. Lurie Children’s Hospital, Chicago, Illinois; 7 Department of Medicine, Northwestern University Feinberg School of Medicine, Chicago, Illinois

## Abstract

This cross-sectional study examines the associations of telework during the COVID-19 pandemic with parents’ general health, changes to mental health, and parenting stress.

## Introduction

The COVID-19 pandemic changed work arrangements with more frequent work from home, or *telework*. Studies^1,2^ conducted before COVID-19 have found conflicting associations between telework and employee health. Parents are a unique subset of employees, yet little is known about how telework is related to the health of working parents.^1^ The purpose of this study was to examine the associations of telework during the COVID-19 pandemic with parents’ general health, changes to mental health, and parenting stress.

## Methods

Survey data for this cross-sectional study were collected from May to July 2022 through a panel survey from all 77 neighborhoods in Chicago, Illinois, a sociodemographically diverse urban center. Eligibility criteria included being aged 18 years or older and the parent of 1 or more child living in the household. Respondents were recruited from probability samples (43.1% completion rate; 596 of 1382 eligible invitees) and nonprobability samples (eAppendix in Supplement 1). The institutional review board at Lurie Children’s Hospital approved the study, and participants provided informed consent electronically. This study followed the STROBE reporting guideline. Full details of the methods and statistical analysis are shown in the eAppendix and eTable in Supplement 1.

## Results

Among the 1060 parent respondents, 825 (74.3%) who were currently employed composed our analytic sample. Among employed parents, 599 (52.5%) were female and 548 (62.5%) teleworked. A higher proportion of teleworking parents were White (244 parents [45.0%]), vs Black (99 parents [14.6%]) or Hispanic (145 parents [28.5%]); conversely, a higher proportion of on-site working parents were Black (84 parents [26.0%]) and Hispanic (98 parents [41.9%]) than White (71 parents [23.8%]) (Table 1). After adjustment, those who teleworked had increased odds of parenting stress compared with on-site work (adjusted odds ratio [aOR], 1.88; 95% CI, 1.20-2.93), but no difference in general health status (aOR, 1.23; 95% CI, 0.78-1.93) or improved mental health (aOR, 1.14; 95% CI, 0.64-2.04) (Table 2). Teleworking fathers reported higher parenting stress than on-site working fathers (aOR, 2.33; 95% CI, 1.03-5.35), but there was no association for mothers (aOR, 1.53; 95% CI, 0.93-2.49).

**Table 1.** Sample Characteristics of Working Parents, by On-Site Work and Telework

Characteristic	Parents, unweighted No. (weighted %)			*P* value^a^
	Total (N = 825 [100.0])	On-site work (n = 277 [37.5])	Telework (n = 548 [62.5])	
Age, y (n = 825)				
<35	333 (31.7)	137 (39.8)	196 (26.9)	.005
≥35	492 (68.3)	140 (60.2)	352 (73.1)	
Gender (n = 821)				
Female (mothers)	599 (52.5)	211 (56.3)	388 (50.3)	.26
Male (fathers)	222 (47.5)	65 (43.7)	157 (49.7)	
Race and ethnicity (n = 825)				
Black, non-Hispanic	183 (18.9)	84 (26.0)	99 (14.6)	<.001
Hispanic	243 (33.5)	98 (41.9)	145 (28.5)	
White, non-Hispanic	315 (37.0)	71 (23.8)	244 (45.0)	
Other, multiracial, non-Hispanic^b^	84 (10.56)	24 (8.4)	60 (11.9)	
Income, % of federal poverty level (n = 825)				
<100	74 (8.4)	34 (11.6)	40 (6.5)	<.001
100-399	384 (48.1)	167 (63.3)	217 (39.1)	
≥400	367 (43.5)	76 (25.1)	291 (54.4)	
General parental health (n = 824)				
Excellent or very good	529 (64.0)	161 (57.9)	368 (67.7)	.049
Good, fair, or poor	295 (36.0)	116 (42.1)	179 (32.3)	
Improved mental health (n = 814)				
Yes	152 (19.5)	47 (18.6)	105 (19.9)	.74
No	662 (80.5)	226 (81.4)	436 (80.1)	
Parenting stress (n = 822)				
All or most of the time	398 (35.1)	91 (27.3)	207 (39.7)	.008
Some of the time or never	524 (64.9)	185 (72.7)	339 (60.3)	

^a^ Computed with χ^2^ test between categories.

^b^ The other, multiracial non-Hispanic category includes those self-identifying as Chinese, Filipino, Japanese, Korean, Vietnamese, Asian Indian, Samoan, Guamanian or Chamorro, Native Hawaiian, Other Pacific Islander, America Indian or Alaska Native, and some other race.

**Table 2.** Associations of Telework and General Health Status, Improved Mental Health and Parenting Stress Among Working Mothers and Fathers

Health outcome	Telework option over the last 2 y^a^			
	Unadjusted OR (95% CI)	*P* value	Adjusted OR (95% CI)^b^	*P* value
General health status^c^				
Overall	1.52 (1.00-2.32)	.05	1.23 (0.78-1.93)	.38
Mothers	1.29 (0.80-2.07)	.30	1.07 (0.65-1.76)	.81
Fathers	1.81 (0.85-3.82)	.12	1.70 (0.72-3.99)	.22
Improved mental health^d^				
Overall	1.09 (0.64-1.86)	.75	1.14 (0.64-2.04)	.65
Mothers	0.72 (0.37-1.39)	.33	0.83 (0.42-1.64)	.58
Fathers	1.38 (0.60-3.16)	.44	1.72 (0.68-4.35)	.26
Parenting stress^e^				
Overall	1.75 (1.14-2.67)	.009	1.88 (1.20-2.93)	.006
Mothers	1.46 (0.92-2.34)	.11	1.53 (0.93-2.49)	.09
Fathers	2.21 (1.02-4.77)	.04	2.33 (1.03-5.25)	.04

^a^ Telework option is compared with on-site work as the reference category.

^b^ Adjusted for age, race and ethnicity, gender, and income as percentage of federal poverty level.

^c^ Refers to very good or excellent general health compared with good or fair health as reference category.

^d^ Refers to better mental health since the start of the COVID-19 pandemic compared with no change or worse as reference category.

^e^ Refers to parenting is stressful always or most of the time compared with some of the time or never as reference category.

## Discussion

In this cross-sectional study, parents who teleworked during the COVID-19 pandemic report higher parental stress compared with parents who worked on-site. Otherwise, our findings were consistent with studies^1,2^ conducted before COVID-19 that found conflicting or no associations between telework and employees’ self-reported health. Our results differed by parent gender: teleworking fathers report higher parenting stress than on-site working fathers, whereas mothers’ parenting stress did not differ by teleworking status. For parents, especially fathers, telework during the COVID-19 pandemic offered a new opportunity to spend time with their children.^3^ Our findings suggest teleworking may also add to parenting stress or that parents with more stressful parenting situations preferentially select telework arrangements. Strategies to support parents who telework, such as promoting work schedule autonomy and employee assistance programs,^2^ may have important health implications for parents and children.

Study limitations include generalizability and a lack of objective health data. The study period was limited to the first 2 years of the pandemic, which included 13 months of remote instruction in Chicago schools,^4^ leading to many parents managing work and schooling at home. Legislation has been proposed to both expand^5^ and limit^6^ telework options. Therefore, it is crucial that researchers, health professionals, and policymakers continue to assess the associations among telework, parenting stress, and parent health.

## Notes

Supplement 1. **eAppendix.** Parent Panel Survey, Recruitment, and Analysis
eReferences
**eTable.** Survey Instrument and Variable Categorization

Supplement 2.
Data Sharing Statement
