# Supplementary material for: Teleworking, Parenting Stress, and the Health of Mothers and Fathers
Source: JAMA Netw Open. 2023 Nov 3;6(11):e2341844. doi: 10.1001/jamanetworkopen.2023.41844 (PMC10625030; doi:10.1001/jamanetworkopen.2023.41844)
Supplement: Supplement 1. — eAppendix. Parent Panel Survey, Recruitment, and Analysis eReferences eTable. Survey Instrument and Variable Categorization [file jamanetwopen-e2341844-s001.pdf]

Supplemental Online Content

Parker JJ, Garfield CF, Simon CD, et al. Teleworking, parenting stress, and the health of mothers and fathers. *JAMA Netw Open*. 2023;6(11):e2341844.
doi:10.1001/jamanetworkopen.2023.41844

eAppendix. Parent Panel Survey, Recruitment, and Analysis
eReferences
eTable. Survey Instrument and Variable Categorization

This supplemental material has been provided by the authors to give readers additional information about their work.

© 2023 Parker JJ et al. *JAMA Network Open.*

eAppendix. Parent Panel Survey, Recruitment, and Analysis

Measures and Analysis

Questions included in the survey related to parents' telework frequency, general health, mental health, and parenting stress (eTable). Self-reported demographic characteristics were collected. Self-reported parent race and ethnicity were categorized as non-Hispanic Black (hereinafter Black), Hispanic or Latinx (hereinafter Hispanic), non-Hispanic White (hereinafter White) and other, multiracial, non-Hispanic. The other, multiracial non-Hispanic category includes those self-identifying as Chinese, Filipino, Japanese, Korean, Vietnamese, Asian Indian, Samoan, Guamanian or Chamorro, Native Hawaiian, Other Pacific Islander, America Indian or Alaska Native, and some other race. Race and ethnicity were analyzed to identify differences in teleworking availability by race and ethnicity. All proportions and analyses were population-weighted; 2-5 sided $P < .05$ was considered significant. Differences in telework by parental characteristics were examined using χ^2 tests. Multivariable logistic regression examined the likelihood of having excellent or very good general parental health, improved mental health since the start of the COVID-19 pandemic, and feelings that parenting stress were present all or most of the time. Covariates included age, race and ethnicity, gender, and income expressed as Federal Poverty Level. Analyses were performed in SAS statistical software version 9.4 (SAS Institute).

Voices of Child Health in Chicago – Parent Panel Survey

The Voices of Child Health in Chicago (VOCHIC) Parent Panel Survey is a triannual survey of Chicago parents about child, adolescent, and family health and well-being. Parents in the panel are from all 77 neighborhoods in Chicago. Parents indicated their preferred method for completing surveys (online or phone) during their initial survey with the panel; all parents completed the survey online and none indicated a preference for a phone survey. Web-based surveys have increased in popularity over the last decade as phone survey response rates have declined.[1] Respondents were compensated \$5 to \$15, based on whether they were first-time participants.

Recruitment and Sampling Methodology

Parent respondents were recruited to participate in this wave of the Voices of Child Health in Chicago (VOCHIC) Parent Panel through one of three mechanisms implemented by NORC at the University of Chicago: 1) the VOCHIC probability-based Parent Panel, 2) NORC's probability-based AmeriSpeak panel, and 3) established online nonprobability survey panels (Dynata and Lucid panels). Responses from nonprobability samples were included to ensure sufficient sample size, which has been shown to be a cost-effective method to supplement probability-based samples.[2,3]

Data Weighting and Analysis

For the probability-based samples, base sampling weights are adjusted to account for nonresponse via a raking ratio method to American Community Survey (2018 ACS PUMS) 18+ Chicago parents population totals associated with the following topline socio-demographic characteristics: age, sex, education, race/ethnicity, and Chicago Community Side, and the following socio-demographic interactions: age x gender, age x race/ethnicity, and race/ethnicity x gender.

For the nonprobability samples, we explicitly account for potential bias using NORC's True North calibration,[2] a hybrid calibration approach developed at NORC based on small area estimation methods. First, the nonprobability sample is raked to the same topline socio-demographic characteristics as the probability sample. Second, the weighted probability sample and the calibrated nonprobability sample were used to develop a small area model to support domain-level estimates, where the domains were defined by race/ethnicity, age, and gender. Finally, the combined AmeriSpeak and nonprobability sample weights were derived such that for the combined sample, the weighted estimate reproduced the small domain estimates (derived using the small area model) for key survey variables.[3] The purpose of TrueNorth calibration is to adjust the weights for the nonprobability sample to bring weighted distributions of the nonprobability sample in line with the population distribution for characteristics correlated with the survey variables. Such calibration adjustments help to reduce potential bias, yielding more accurate population estimates. Pairwise deletion was used for missing data.

eReferences

1. Olson K, Smyth JD. *Report of the AAPOR Task Force on Transitions from Telephone Surveys to Self-Administered and Mixed-Mode Surveys*. 2019. https://aapor.org/wp-content/uploads/2022/11/Report-of-the-Task-Force-on-Transitions-from-Telephone-Surveys-FULL-REPORT-FINAL.pdf

© 2023 Parker JJ et al. *JAMA Network Open.*

2. NORC at the University of Chicago. NORC's TrueNorth Calibration tool for probability and nonprobability samples: New Version 2.0 even more effective. August 2021
3. Yang M, Ganesh N, Mulrow E, Pineau VJ. Evaluating Estimation Methods for Combining Probability and Nonprobability Samples through a Simulation Study. Proceedings of the 2019 Joint Statistical Meeting. Denver, CO. Available at: http://www.asasrms.org/Proceedings/y2019/files/1199569.pdf

eTable: Survey Instrument and Variable Categorization

**Variable**	**Question**	**Categorization**
Telework (work from home)	In your job, over the past 2 years how often have you worked from home or teleworked?	1. Telework = "less than half of the time"/"half"/" "more than half"/"always"<br>2. Onsite-work = "could not work from home" or "Never, I chose to work on the job site"
General Parental Health	In general, how would you rate your health?	1. "excellent"/"very good"<br>2. "good"/"fair"/"poor
Improved Mental Health	Compared to before the COVID-19 pandemic, is your mental health better, the same or worse?	1. Yes = "better"<br>2. No = "no change"/"worse"
Parenting Stress	Indicate how often you feel parenting is stressful	1. "always"/"most of the time"<br>2. "some of the time"/"never."
Race and ethnicity	Please check one or more categories below to indicate what race or races you consider yourself to be; This question is about Hispanic ethnicity. Are you of Spanish, Hispanic, or Latino descent?	1. Black= Black, non-Hispanic<br>2. Hispanic<br>3. White= White, non-Hispanic<br>4. Other/Multi-race, non-Hispanic

© 2023 Parker JJ et al. *JAMA Network Open.*
