# Supplementary material for: Teleworking, Parenting Stress, and the Health of Mothers and Fathers
Source: JAMA Netw Open. 2023 Nov 3;6(11):e2341844. doi: 10.1001/jamanetworkopen.2023.41844 (PMC10625030; doi:10.1001/jamanetworkopen.2023.41844)
Supplement: Supplement 2. — Data Sharing Statement [file jamanetwopen-e2341844-s002.pdf]

Data Sharing Statement

Parker. Teleworking, Parenting Stress, and the Health of Mothers and Fathers. *JAMA Netw Open*. Published November 07, 2023. doi:10.1001/jamanetworkopen.2023.41844

Data
Data available: No

Additional Information
Explanation for why data not available: To protect participant privacy individual patient data will not be available. However, summary data and the data dictionary will be available on an Open Science Framework page.
